# Transcriptional Repression of Hox Genes by *C. elegans* HP1/HPL and H1/HIS-24

**DOI:** 10.1371/journal.pgen.1002940

**Published:** 2012-09-13

**Authors:** Maja Studencka, Radosław Wesołowski, Lennart Opitz, Gabriela Salinas-Riester, Jacek R. Wisniewski, Monika Jedrusik-Bode

**Affiliations:** 1 Department of Genes and Behavior, Epigenetics in C. elegans Group, Max Planck Institute for Biophysical Chemistry, Göttingen, Germany; 2 DNA Microarray Facility, Georg-August University, Göttingen, Germany; 3 Department of Proteomics and Signal Transduction, Max Planck Institute for Biochemistry, Martinsried, Germany; Stanford University, United States of America

## Abstract

Elucidation of the biological role of linker histone (H1) and heterochromatin protein 1 (HP1) in mammals has been difficult owing to the existence of a least 11 distinct H1 and three HP1 subtypes in mice. *Caenorhabditis elegans* possesses two HP1 homologues (HPL-1 and HPL-2) and eight H1 variants. Remarkably, one of eight H1 variants, HIS-24, is important for *C. elegans* development. Therefore we decided to analyse in parallel the transcriptional profiles of HIS-24, HPL-1/-2 deficient animals, and their phenotype, since *hpl-1*, *hpl-2*, and *his-24* deficient nematodes are viable. Global transcriptional analysis of the double and triple mutants revealed that HPL proteins and HIS-24 play gene-specific roles, rather than a general repressive function. We showed that HIS-24 acts synergistically with HPL to allow normal reproduction, somatic gonad development, and vulval cell fate decision. Furthermore, the *hpl-2*; *his-24* double mutant animals displayed abnormal development of the male tail and ectopic expression of *C. elegans* HOM-C/Hox genes (*egl-5* and *mab-5*), which are involved in the developmental patterning of male mating structures. We found that HPL-2 and the methylated form of HIS-24 specifically interact with the histone H3 K27 region in the trimethylated state, and HIS-24 associates with the *egl-5* and *mab-5* genes. Our results establish the interplay between HPL-1/-2 and HIS-24 proteins in the regulation of positional identity in *C. elegans* males.

## Author Summary

Linker histone (H1) and heterochromatin protein 1 (HP1) play central roles in the formation of higher-order chromatin structure and gene expression. Recent studies have shown a physical interaction between H1 and HP1; however, the biological role of histone H1 and HP1 is not well understood. Additionally, the function of HP1 and H1 isoform interactions in any organism has not been addressed, mostly due to the lack of knockout alleles. Here, we investigate the role of HP1 and H1 in development using the nematode *C. elegans* as a model system. We focus on the underlying molecular mechanisms of gene co-regulation by H1 and HP1. We show that the loss of both HP1 and H1 alters the expression of a small subset of genes. *C. elegans* HP1 and H1 have an overlapping function in the same or parallel pathways where they regulate a shared target, the Hox genes.

## Introduction

Linker histone H1 and heterochromatin protein HP1 are involved in numerous processes ranging from stabilizing heterochromatin condensation to the regulation of gene expression [1]–[5]. As has been reported, a methylation mark on vertebrate histone H1 is specifically recognized by the chromodomain of HP1. However, the exact biological role of HP1 binding to linker histone has not been determined [6].

The functions of HP1 and H1 proteins are mainly dependent on the cell type in which particular variants are expressed. Although the number of H1 (11) and HP1 variants (3) presents difficulties in studying the effect of H1 and HP1 depletion in mice, some data has emerged [3], [7]–[10]. For example, loss of HP1β results in defective development of neuromuscular junctions and the cerebral cortex [10], whereas depletion of three of eleven H1 genes causes lethality connected with a very broad range of defects in mice [11]–[12]. In ES cells, the lack of three somatic H1 variants leads to changes in nucleosome spacing and local chromatin compaction, and this is correlated with decreased levels of H3K27 trimethylation [11]. Additionally, H1 is necessary to establish and maintain the DNA methylation pattern in a subset of genes including the reproductive homeobox (Rhox) gene cluster [13].

*C. elegans* possesses eight linker histone variants and two HP1 homologues, HPL-1 and HPL-2 [14]–[16]. Mutation of *hpl-2* results in defective vulval and germline development at elevated temperatures [15]–[17]. *hpl-1*, in contrast to *hpl-2*, does not have visible effects on *C. elegans* development at different temperatures, however, *hpl-1* acts redundantly with *hpl-2* to control larval development, somatic gonad development and vulval cell fate determination [17]. Our previous study revealed that HPL-1 recognizes the linker histone variant HIS-24 when it is mono-methylated at lysine 14 (HIS-24K14me1), similar to the situation in vertebrates [16]. Additionally, we showed that HIS-24 interacts with H3K27me3 [18]. The H3K27me3 modification correlates with a repressive chromatin state that inhibits expression of many developmentally regulated genes. This is consistent with studies of Hox loci demonstrating that enrichment of H3K27me3 recruits the binding of Polycomb group proteins (PcG) [19].

The Hox genes encode conserved homeodomain-containing transcription factors that control the positional identities of cells along the anterior–posterior axis [20]–[21]. The expression pattern of Hox genes appears to be regulated by two evolutionarily conserved PcG complexes, the ESC/E(Z) complex and the PRC1 complex. Both have been identified in flies and mammals and are linked to modulation of repressive chromatin structures [21]. The *C. elegans* Hox cluster consisting of *lin-39*, *ceh-13*, *mab-5* and *egl-5* (orthologs of *Drosophila Scr*, *labial*, *ftz* and *Abd-B*, respectively) is quite degenerated in comparison to Hox clusters in other species [22] but, as in mammals, is also globally repressed by Polycomb group (PcG) proteins [20], [23]. Mutations in *mes-2* and *mes-6*, which encode the *C. elegans* ESC/E(Z) complex, result in ectopic expression of Hox genes [23]. A similar phenotype has also been observed in the absence of *sop-2* or *sor-1* genes. SOP-2 and SOR-1 form another *C. elegans* PcG-like complex which shares many structural and functional properties with the *Drosophila* PRC1, and is involved in the global repression of Hox gene expression. Loss of *sop-2* and *sor-1* results in gross homeotic transformations [24]–[25].

To elucidate the function of H1 and HP1 related proteins in *C. elegans*, we decided to generate double and triple mutants, since *hpl-1*, *hpl-2* and *his-24* deficient nematodes are viable, and since HIS-24K14me1 is recognized by HPL-1 [16]–[17], [26]. We performed global transcriptional analyses of single, double and triple mutant animals, and we found that HPL-1/-2 and HIS-24 regulate a relatively small number of genes. We provide evidence that the methylated form of HIS-24 (HIS-24K14me1) and HPL-2 are involved in the regulation of *mab-5* and *egl-5* expression by binding to H3K27me3, although HIS-24K14me1 does not interact with HPL-2 [16]. Furthermore, we observed that HIS-24 and HPL-2 act in parallel pathway as MES (PcG) proteins, and loss of their activity causes defects of male tail structures. Overall, our data suggest a common and dual role for *C. elegans* H1 and HP1, functioning both as chromatin architectural proteins and at the same time as modifiers of a small subset of genes. Furthermore, we provide the first direct evidence for redundant functions of H1 and HP1 in metazoan development.

## Results

### HP1 and HIS-24 are not global repressors of transcriptional activity in *C. elegans*

**Figure 1:**
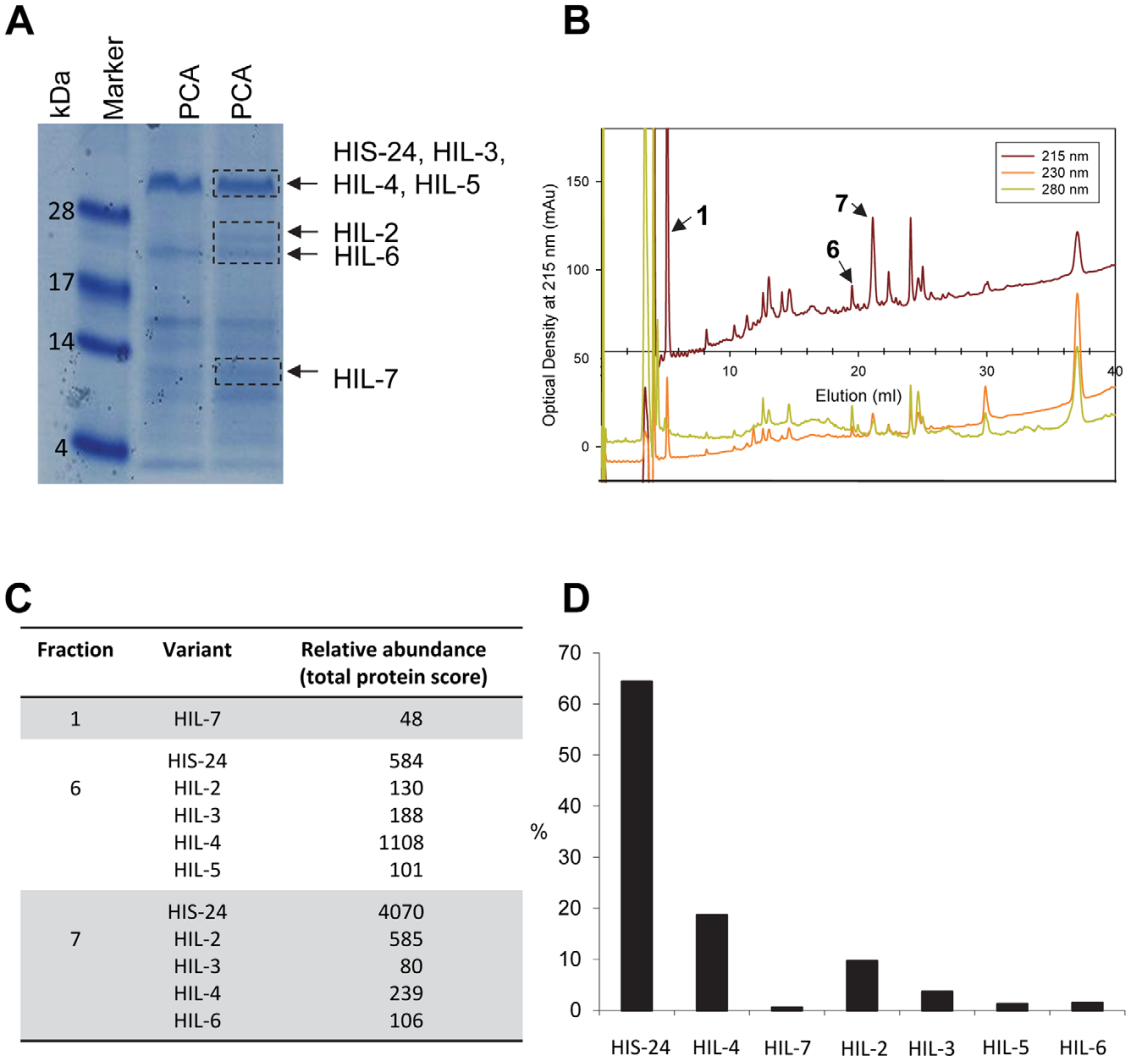
*C. elegans* H1 proteomics. (A) L4 larval stage of wild type animals were extracted with 5% PCA. The PCA extract was chromatographed on C18 column. (B) Fractions 1, 6, and 7 containing H1 were analyzed. (C) Absolute protein abundances of linker histone variants were calculated from spectral counts. The table shows the relative abundance of the variants on the basis on their protein scores (Mascot). The total protein score corresponds directly to the protein abundance, because the histones have similar masses. HIL-1 (H1.X) was found only in an HCl extract (data not shown). (D) Abundance of *C. elegans* H1 variants in percent (%).

*C. elegans* contains two related HP1 proteins (HPL) and eight linker histone variants [14]–[15]. Only one of the eight linker histone variants, HIS-24 is important for germline development, with its absence resulting in reduced fertility and de-repression of extrachromosomal transgenic arrays in the germline [14]. As we previously reported, the absence of HIS-24 did not affect protein levels of the other histone variants, in contrast to the mammalian H1 subtypes which are sufficient to compensate for the loss of a single linker histone [7], [16]. Furthermore, we showed that *C. elegans* heterochromatin protein 1 variant, HPL-1 recognizes and binds the methylated form of HIS-24 [16]. Given the physical interaction of HPL-1 with HIS-24 mono-methylated at lysine 14 and their role in chromatin silencing and germline developmental processes [15]–[17], we decided to study HPL and HIS-24 function in transcriptional regulation in *C. elegans*. It was of great interest to determine how the HPL subtypes (HPL-1 and HPL-2) and HIS-24 affect gene expression. To determine the contribution of HIS-24 and HPL-1/-2 to the control of gene transcription, we compared the gene-expression profiles of single null mutations in the *hpl-1*, *his-24* and *hpl-2* as well as profiles of *hpl-1his-24*, and *hpl-2*; *his-24* double, and *hpl-2*; *hpl-1his-24* triple mutant animals in L4 larval stages grown at 21°C. We decided to use L4 larval stages because HIS-24 is the most abundant linker histone H1 variant at this stage according to mass spectrometry-based protein expression data (Figure 1).

**Figure 2:**
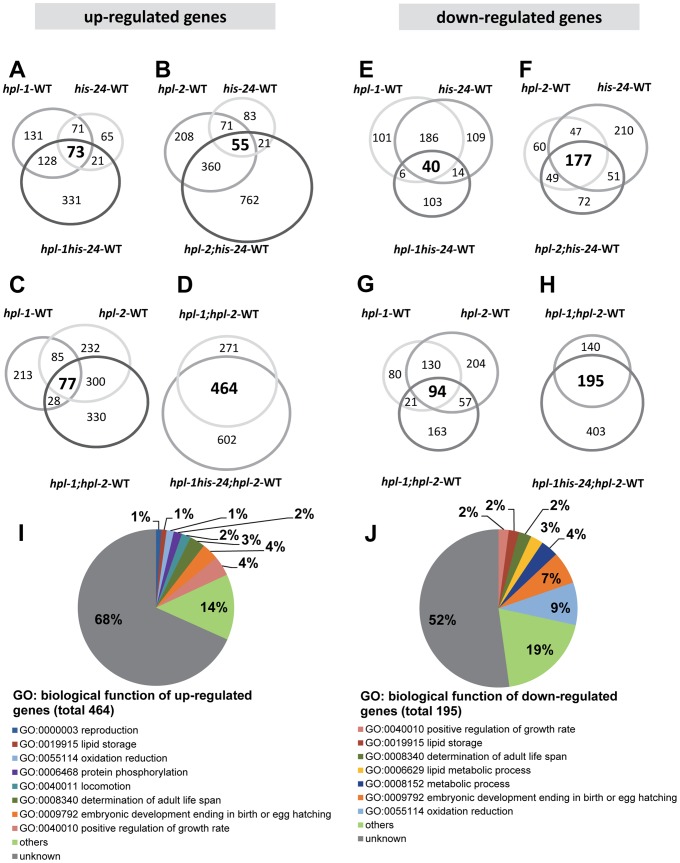
HPL and HIS-24 are not global repressors of transcriptional activity. (A–H) Venn diagrams on the basis of whole genome microarray (16278 genes) showing the extent of overlap among genes that are associated with HIS-24 and HPLs activity. Values for fold change are the average from 2 independent biological replicates (FDR<0.05; |log_2_-fold change|>1.5). WT-wild type; significant overlapping among the candidate lists - p-value<0.000001 for all pair-wise comparisons by hypergeometric tests. (I, J) Functional clusters of HPL-1, HPL-2 and HIS-24 co-regulated genes in *C. elegans*. The molecular functions and the percentage of the total for each group are indicated. Gene Ontology (GO) terms taken from WormBase (http://www.wormbase.org).

By microarray we observed very few changes in the gene expression profiles of either single, double, or triple mutants when compared with wild type animals at L4 larval stages. Among the 16,278 target probe sets assayed, we identified only modest changes in expression of just a small number of genes (Figure 2A–2H, Table 1). The majority of genes exhibiting changes were upregulated (6.5%) in the absence of the three heterochromatin components HIS-24, HPL-1 and HPL-2, in contrast to 3.7% downregulated genes from a total of 16,278 genes analyzed (FDR<0.05) suggesting that HPL-1/-2 and HIS-24 are not global repressors of transcriptional activity (Table 1). The deletion of both *hpl-1* and *hpl-2* genes caused up-regulation of 4.5% genes and downregulation of 2.1% of a total 16,278 genes when compared to wild type (WT) animals.

**Table 1:** Summary of agilent microarray analysis of gene expression in mutant animals versus wild type (16, 278 genes analysed).

Genotype	Up-genes (%)	Down-genes (%)
	FDR<0.05; |log_2_-fold change|>1.5 (total)	
*his-24(ok1024)*	1.4	1.9
*hpl-1(tm1624)*	2.5	2.0
*hpl-2(tm1489)*	4.2	1.9
*hpl-1(tm1624); hpl-2(tm1489)*	4.5	2.1
*hpl-1(tm1624) his-24(ok1024)*	3.4	1.0
*hpl-2(tm1489); his-24(ok1024)*	7.2	2.1
*hpl-1(tm1624) his-24(ok1024); hpl-2(tm1489)*	6.5	3.7

As previously reported, HPL-2 binds to HIS-24K14me1 through its association with HPL-1, and the heterochromatin proteins HPL-1 and HPL-2 play redundant roles in *C. elegans* development [16]–[17]. Considering these observations we compared transcriptional profiles between *hpl-2 (tm1489); hpl-1(tm1624)* double mutants and *hpl-2 (tm1489); hpl-1(tm1624) his-24(ok1024)* triple mutant animals. We found that 464 up-regulated (2.9% of 16,278) and 195 down-regulated (1.2% of 16,278) genes were commonly affected (FDR<0.05; p-value<0.000001 for all pair-wise comparisons by hypergeometric tests) (Figure 2, Table S1). Among the 464 up-regulated genes we identified some significantly enriched in GO terms associated with growth regulation (Fisher exact test (FET) *P* = 4×10^−6^), determination of adult life span (FET *P* = 2×10^−6^), locomotion (FET *P* = 0,003), protein phosphorylation (FET *P* = 0,04), reproduction (FET *P* = 0,05) and lipid storage (FET *P* = 0,05). The 195 genes that are down-regulated are enriched in GO terms associated with oxidation reduction (FET *P* = 0,003), embryonic development (FET *P* = 0,002) and metabolic process (FET *P* = 0,04). We identified common response proteins including heat shock proteins (HSP-12.3, -12.6, -16.2 and -17), enzymes (cytochromes) of the P450 family involved in protection against toxins (CYP-13A12, CYP-33C4, CYP-33D3, CYP-34A2, CYP-34A4 and CYP-34A9), metabolic enzymes such as the fatty acid-coenzyme A (CoA) synthetase ACS-1 and the fatty acid/retinol binding proteins FAR-5, -7 (Table S1). Furthermore, we observed the induction of oxidative stress proteins such as glutathione *S*-transferases (GST) and genes commonly associated with increased stress resistance – for example, the mitochondrial *sod-3* superoxide dismutase gene (Table S1).

In conclusion, deletion of the different HPL variants and HIS-24 caused an alteration in the expression of a limited number of genes, different in each HPL variant and HIS-24. Most of the genes are affected by a single HPL variant and HIS-24, supporting the theory that HPL isoforms or HIS-24 play specific roles in gene expression. Nonetheless, a proportion of genes are altered by more than one HPL variant as well as HIS-24, suggesting redundant roles for HIS-24 and HPL variants, and for HPL-1/-2 may also exist.

### HIS-24 acts synergistically with HPL proteins to allow normal reproduction, somatic gonad development, and vulval cell fate decisions

**Figure 3:**
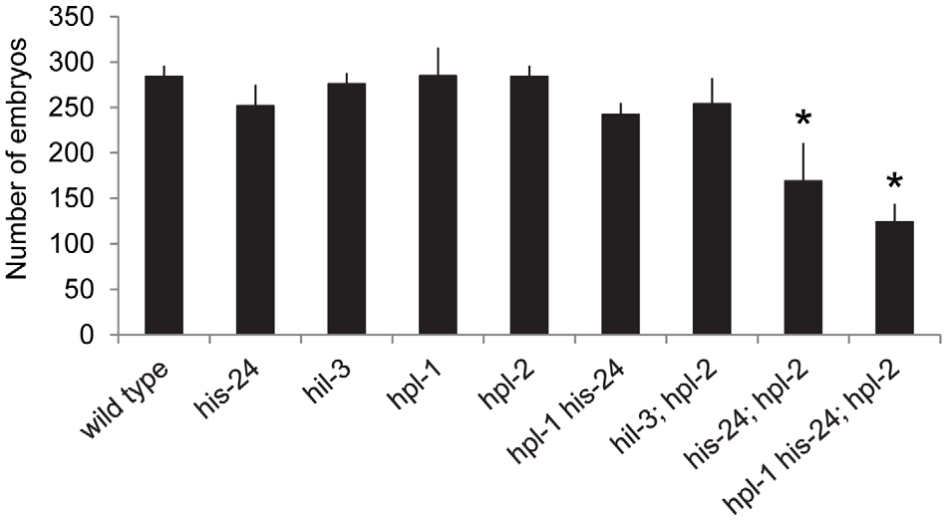
HIS-24 act synergistically with the *hpl*-genes to control brood size at 21°C. Number of embryos (± SEM), 20 animals scored: * p<0.0001, vs. wild type.

**Figure 4:**
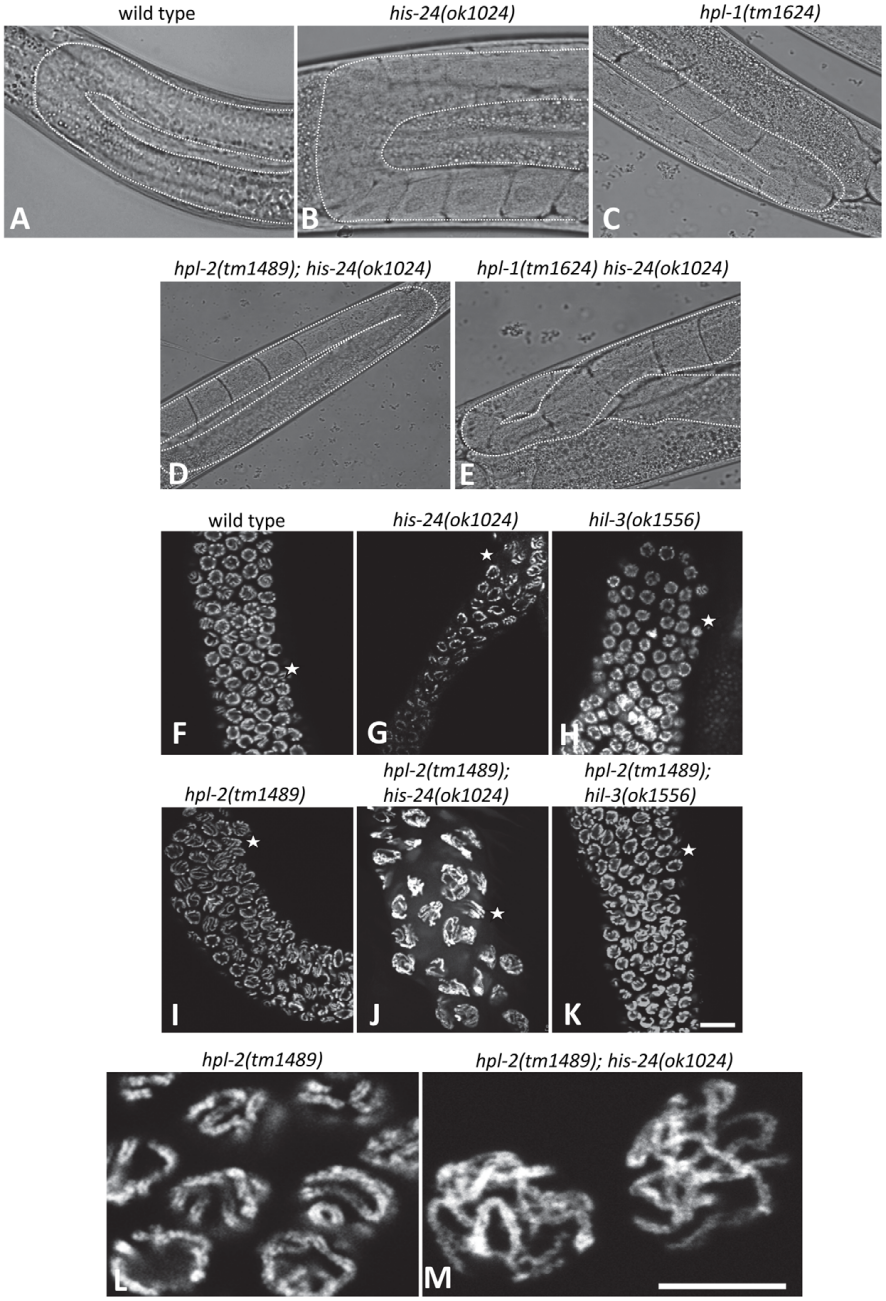
HIS-24 and HPL-1/-2 depletion results in abnormal somatic gonad development and reduction of chromatin compaction. (A–D) Normal U-shaped form of gonad arm of wild type, *his-24* or *hpl-1* single, and *hpl-2; his-24* double mutants. (E) *hpl-1his-24* mutant animal demonstrates aberrant loop form of gonad arm. (F–K) DAPI stained gonad arms of *his-24*, *hil-3* or *hpl-2* single and *hpl-2; his-24* or *hpl-2; hil-3* double mutants. Stars point to pachytene stage germ nuclei. Scale bar: 10 µm. (L, M) Morphology of pachytene stage nuclei of the germ line of *hpl-2* mutant animals compared to the nuclei of *hpl-2; his-24* double mutants. Scale bar: 7.5 µm.

In parallel to microarray analysis we investigated the biological role of HIS-24 and HPL proteins in *C. elegans*. For morphological defects we scored *hpl-1(tm1624) his-24(ok1024)*, and *hpl-2(tm1489); his-24(ok1024)* double mutants as well as *hpl-2(tm1489); hpl-1(tm1624) his-24(ok1024)* triple mutant animals. In particular, we focused on germline nuclei morphology, hermaphrodite vulval development and the somatic patterning of the male tail since these tissues are known to be affected by mutations in chromatin factors, and HPL-2 influences vulval cell fate specification in the synMuv (synthetic multivulva) pathway [14]–[15], [27]. We found that the deletion of *hpl-2(tm1489)* together with *his-24(ok1024)* results in synergistic non-lethal defects of vulval cell fate specification (everted vulva, multivulva) and sterility at 21°C, and at 25°C (Table 2). While the observed phenotypic effects at 21°C were minor in contrast to the situation at 25°C, it is tempting to speculate that the effects can be also modulated through unknown mechanisms, environmental cues (temperature), which in itself may also lead to significant side-effects. Additionally, decreased brood sizes were observed in *hpl-2(tm1489); his-24(ok1024)* double and *hpl-2(tm1489); hpl-1(tm1624) his-24(ok1024)* triple mutant animals grown at 21°C (Figure 3). The brood size of the *hpl-2(tm1489); his-24(ok1024)* was strongly decreased by 35% of wild type worms, and was further decreased to about 50% in the *hpl-2(tm1489); hpl-1(tm1624) his-24(ok1024)* triple mutant animals (Figure 3). These results were consistent with our microarray data analysis that revealed differential expression of genes involved in the embryonic development or reproduction (Table S1). Furthermore, we observed several defects in the morphology of the somatic gonad of *hpl-1(tm1624) his-24(ok1024)* double mutant animals grown at 21°C. In wild type, single mutant and *hpl-2(tm1489); his-24(ok1024)* double mutant the gonad arms form an U-shaped structure (Figure 4A–4D). In contrast, in the double mutant *hpl-1(tm1624) his-24(ok1024)* 25% of gonad arms (161 of 642) form a loop (Figure 4E). These results suggest that both proteins HIS-24 and HPL-1 are involved in the somatic gonad development whereas HIS-24 and HPL-2 influence vulva cell fate specification and reproduction (Table 2).

**Table 2:** Genetic interaction of *hpl-1*, *hpl-2*, and *his-24*.

Genotype	% sterility		% multivulva		% everted vulva	
	at 21°C	at 25°C	at 21°C	at 25°C	at 21°C	at 25°C
wild type	0 (320)	0 (330)	0 (320)	0 (330)	0 (320)	0 (330)
*his-24(ok1024)*	7 (257)	8 (235)	0 (257)	0 (235)	0 (257)	0 (235)
*hpl-1(tm1624)*	0 (401)	0 (420)	0 (401)	0 (420)	0 (401)	0 (420)
*hpl-2(tm1489)*	5 (356)	66 (424)	5 (356)	22 (424)	0 (356)	32 (424)
*hpl-1(tm1624) his-24(ok1024)*	0 (321)	0 (326)	0 (321)	0 (326)	0 (321)	2 (326)
*hpl-2(tm1489); his-24(ok1024)*	11 (881)	88 (528)	9 (881)	28 (528)	12 (881)	62 (528)
*hpl-1(n4317);hpl-2(tm1489)*	0 (453)	L2/L3 arrest	4 (453)	L2/L3 arrest	5 (453)	L2/L3 arrest
*hpl-1(tm1624) his-24(ok1024); hpl-2(tm1489)*	11 (729)	L2/L3 arrest	12 (729)	L2/L3 arrest	28 (729)	L2/L3 arrest

The mutant animals show temperature sensitive phenotypes.

### HIS-24 and HPL-2 are required for chromatin compaction

Since HPL-2 and HIS-24 are required for germline development and for the chromatin based germline-specific silencing mechanism [14]–[15], [26], we asked whether they influence the structure of nuclei. In-depth analysis revealed that the germline nuclei of *hpl-2(tm1489)*; *his-24(ok1024)* double mutants differ in size and morphology when compared to single mutants or to wild type worms grown at 21°C (Figure 4F–4I, 4L). The observed chromatin of 86% of gonad arms (36 of 42) had a more open, relaxed structure suggesting that HIS-24 and HPL-2 play a function in chromatin condensation in the germline (Figure 4J, 4M). To assess the specific requirements for HIS-24 among the H1 isoforms, we also tested *hpl-2(tm1489)*;*hil-3(ok1556)* double mutant strain to determine if the observed changes in the chromatin compaction is linker histone variant specific (Figure 4K). As shown, loss of *hpl-2* and linker histone variant *hil-3* did not cause defects in chromatin compaction in contrast to *hpl-2*; *his-24* strain. In addition, we also did not observe involvement of HPL-2 and HIL-3 on brood size (Figure 3).

To determine if the loss of HIS-24 and HPL proteins also influence chromatin histone modifications as well as core histone H3 level, we performed western blot analysis of mutant animals. No gross changes were observed in the methylation and core histone H3 levels using antibodies directed against H3K9me3, H3K27me3, and H3 (Figure S1). In addition, we did not detect changes in chromatin modification marks on a cellular level by immunofluorescence (data not shown) indicating that the observed effects of chromatin compaction are not correlated with alterations of histone modifications in *hpl-2(tm1489); his-24(ok1024)* double mutant animals.

### Mutations in *his-24* and *hpl-2* cause male tail defects

Loss of *hpl-1*, *-2* and *his-24* function results in changes of transcriptional regulation of genes encoding nuclear hormone receptor family genes (*nhr-60*, *nhr-156*), transcription factors (*miz-1*, *zip-3*, *zip-8*, *madf-2*), homeobox *ceh-82* and homeodomain *lim-7* genes (Table S1). Moreover, *hpl-2* regulates *lin-39* Hox gene expression in vulval precursor cells (VPCs) [27]. Therefore we tested whether *hpl-1*, *-2* and *his-24* genes are involved in the regulation of Hox gene expression during the somatic patterning of the male tail.

**Figure 5:**
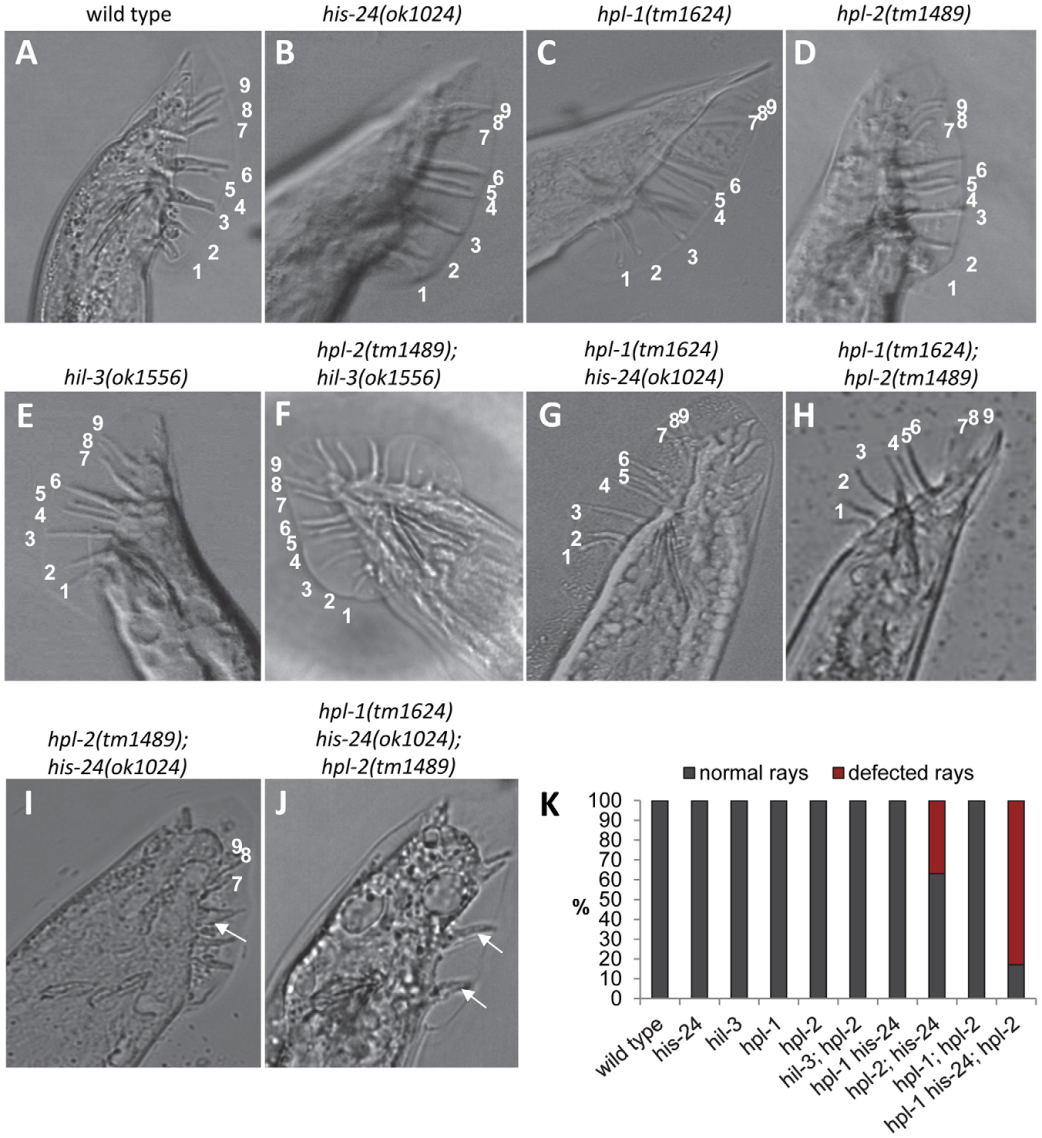
*his-24* and *hpl-2* genes are involved in proper postembryonic development of male-specific structures. (A–F, I–J) All nine rays are formed normally in *his-24*, *hil-3*, *hpl-1* or *hpl-2* single mutants and in *hpl-1his-24* as well as in *hpl-1; hpl-2* and *hpl-2; hil-3* double mutant males. (G, H) Abnormal male tails of *hpl-2*; *his-24* and *hpl-2*; *hpl-1his-24* mutant animals. Arrows point to ray fusions. (K) Quantification of ray defects associated with single, *hpl-2; his-24* double and *hpl-1his-24; hpl-2* triple mutations. The animals were growing on *him-14* (high incidence of males) feeding plates at 21°C.

The wild type male tail possesses nine pairs of bilateral sensory rays that function in locating and mating with hermaphrodites. Normally, the posterior hypodermal blast cells V5 and V6 produce six pairs of rays (ray 1 to ray 6), while the blast cell T gives rise to the three rays (rays 7–9) [23]–[25]. We found that mutations in both *his-24* and *hpl-2* (37%, 51 of 73 males with defected rays) as well as in *his-24*, *hpl-1* and *hpl-2* (83%, 76 of 107 males with defected rays) cause abnormalities in patterning of blast cells V that result in fused and atypical (under-developed) rays, while the single and *hpl-1; hpl-2* and *hpl-1 his-24* double mutations have normal development of rays (Table S2, Figure 5A–5E, 5G). Although *hpl-1* mutation alone or in combination with *his-24* or *hpl-2* had no visible effect on the male tail at 21°C (Figure 5C, 5G–5H), it appeared to be partially redundant in combination with *hpl-2* and *his-24* double mutations. As Figure 5J and Table S2 show, the number of under-developed rays is significantly increased (up to 42%, 39 of 107 males) in the *hpl-2(tm1489); hpl-1(tm1624) his-24(ok1024)* triple mutant compared to the *hpl-2(tm1489); his-24(ok1024)* double mutant males (13%, 18 of 73 males) (Figure 5I). This synergism suggests that *hpl-1* only in combination with *his-24* and *hpl-2* plays functions in the patterning of the male tail. We also tested *hil-3; hpl-2* double mutant animals for the mail tail phenotype. We did not observe any defects in the patterning of the male tail of *hil-3; hpl-2* double mutant animals in contrast to *hpl-2*; *his-24* animals suggesting that HIS-24 (in combination with HPL-2) specifically affects the patterning of the mating structures in *C. elegans* (Figure 5F–5I).

### HIS-24 and HPL-2 are required for inhibiting the ectopic expression of *mab-5* and *egl-5* Hox genes

In agreement with previous observations we analyzed the ability of *his-24*, *hpl-1* and *hpl-2* genes to regulate *mab-5* and *egl-5* expression [28]–[29]. Interestingly, these two Hox genes are required for V ray development [23] and *mab-5* was slightly up-regulated in our microarray analysis of *hpl-2(tm1489); hpl-1(tm1624) his-24(ok1024)* mutant animals (data not shown).

**Figure 6:**
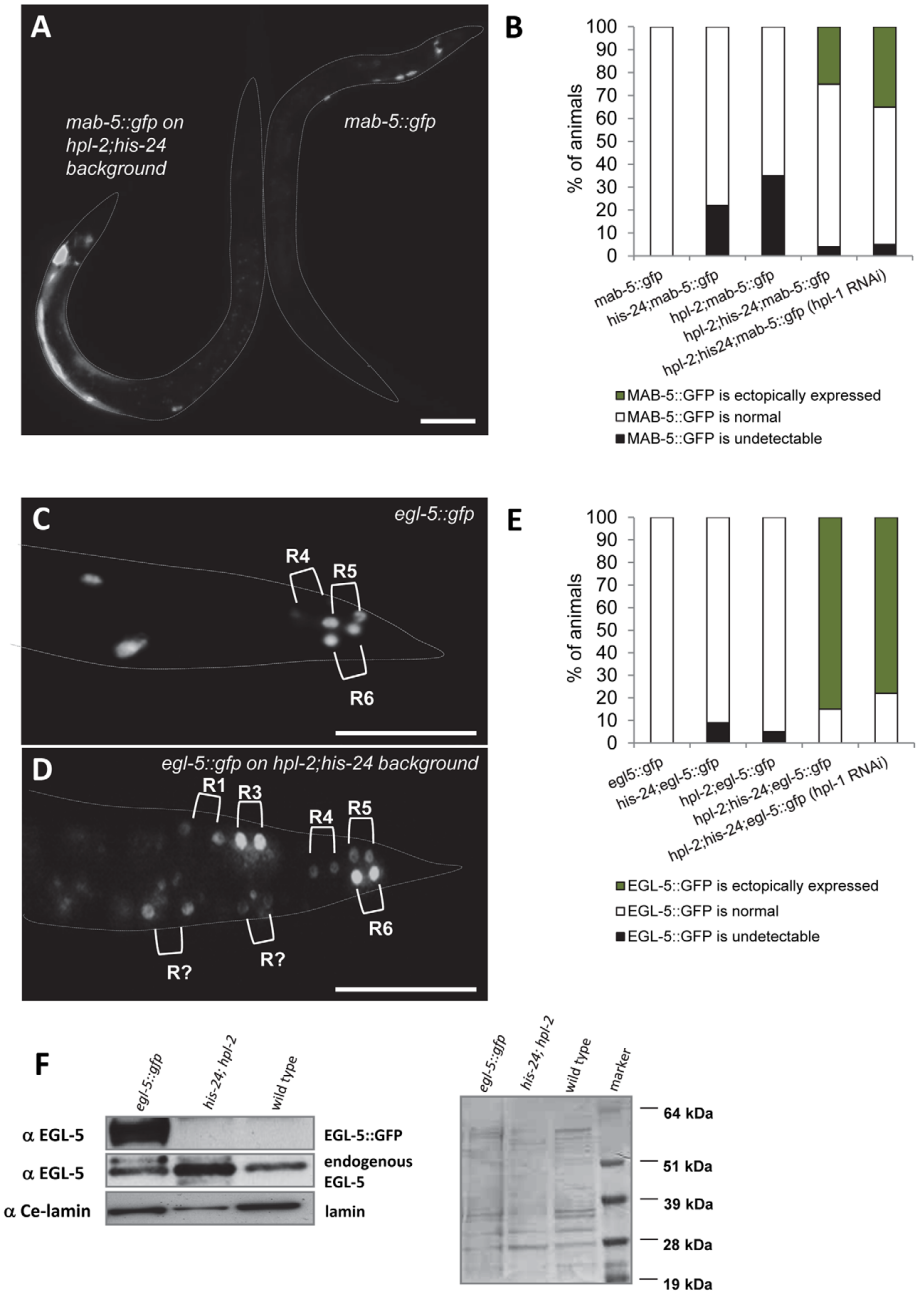
*mab-5* and *egl-5* Hox genes are ectopically expressed in *hpl-2*; *his-24* mutants. (A) A *hpl-2*; *his-24* early L3 mutant male ectopically expresses *mab-5::gfp* in hypodermal syncytium cell ((hyp7); animal on the left side). Expression of *mab-5::gfp* in a wild type early L3 male marks very few cells at the posterior (animal on the right side). Scale bar: 25 µm. (B) Quantification of progeny of single and double *hpl-2*; *his-24* mutant hermaphrodites with males carrying *mab-5::gfp* reporter versus wild type males. (C, D) In a wild type L3 male, expression of *egl-5::gfp* is limited to the daughters of the ray precursor cells R4, R5 and R6 which give rise to rays 3–6. In *hpl-2*; *his-24* L3 mutant male the reporter is expressed in additional ray sublineages. Scale bar: 25 µm. (E) Quantification of progeny of single and double *hpl-2*; *his-24* mutant hermaphrodites with males carrying *egl-5::gfp* reporter versus wild type males. (F) Western blot of protein extracts (150 males) from *C. elegans* wild type, *hpl-2*; *his-24* double mutant and *egl-5* transgenic strain probed with antibody raised against EGL-5. Anti-EGL-5 recognized the EGL-5::GFP fusion protein and endogenous EGL-5 in *egl-5::gfp* transgenic strain. Protein loading was confirmed by probing with an anti-Ce-lamin antibody and western blot stained with Ponceau S.

We compared expression of *egl-5::gfp* and *mab-5::gfp* reporter genes in wild type animals and in combination with *his-24(ok1024)*, *hpl-1(tm1624)* and *hpl-2(tm1489)* background mutations (Figure 6). We observed that *mab-5::gfp* reporter is ectopically expressed in approximately 30% of early L3 stage of *hpl-2(tm1489); his-24(ok1024)* double mutant males scored (n = 100) (Figure 6A, 6B). Altered expression of this reporter was also detected in adult males. Similarly, about 80% of L3 stage of *hpl-2(tm1489); his-24(ok1024)* double mutant males (n = 100) displayed ectopic expression of EGL-5::GFP protein in two daughters of ray precursors anterior to R4, R5 and R6 sublineages (Figure 6C–6E). We did not observe any significant enhancement of the ectopic expression of *mab-5* and *egl-5* Hox genes in *hpl-1* depleted *hpl-2(tm1489); his-24(ok1024)* double mutant animals. For the crossing with *mab-5::gfp* transgenic strain we did not use triple mutant animals due to *hpl-1 his-24; hpl-2* phenotype (sterile worms, worms with everted vulva or multivulva; Table 2).

To verify HPL-1 depletion directly and to examine the extent of HPL-1 knockdown we tested the *hpl-1* depleted *hpl-2; his-24* mutant animals for presence of HPL-1 on the western blot. We found that *hpl-1*RNAi strongly reduces HPL-1 level compared to the controls (Figure S2).

Since mutations in *hpl-2* and *his-24* affect transgene expression in *C. elegans* [14]–[15] we assessed the expression level of the endogenous EGL-5 in *hpl-2*; *his-24* double mutant males. Western blot of *hpl-2*; *his-24* double mutant males probed with EGL-5 antibody revealed an increased level of endogenous EGL-5 protein of predicted size (26 kDa) compared to EGL-5 level of wild type *C. elegans* and *egl-5::gfp* transgenic line (Figure 6F) [29]. Altogether, these results suggest that HIS-24 and HPL-2 silence the Hox gene cluster, either by general repression of the transcriptional activity, or through a specific biochemical and structural function in Hox gene silencing.

### HIS-24 binds to *egl-5* and *mab-5* loci

**Figure 7:**
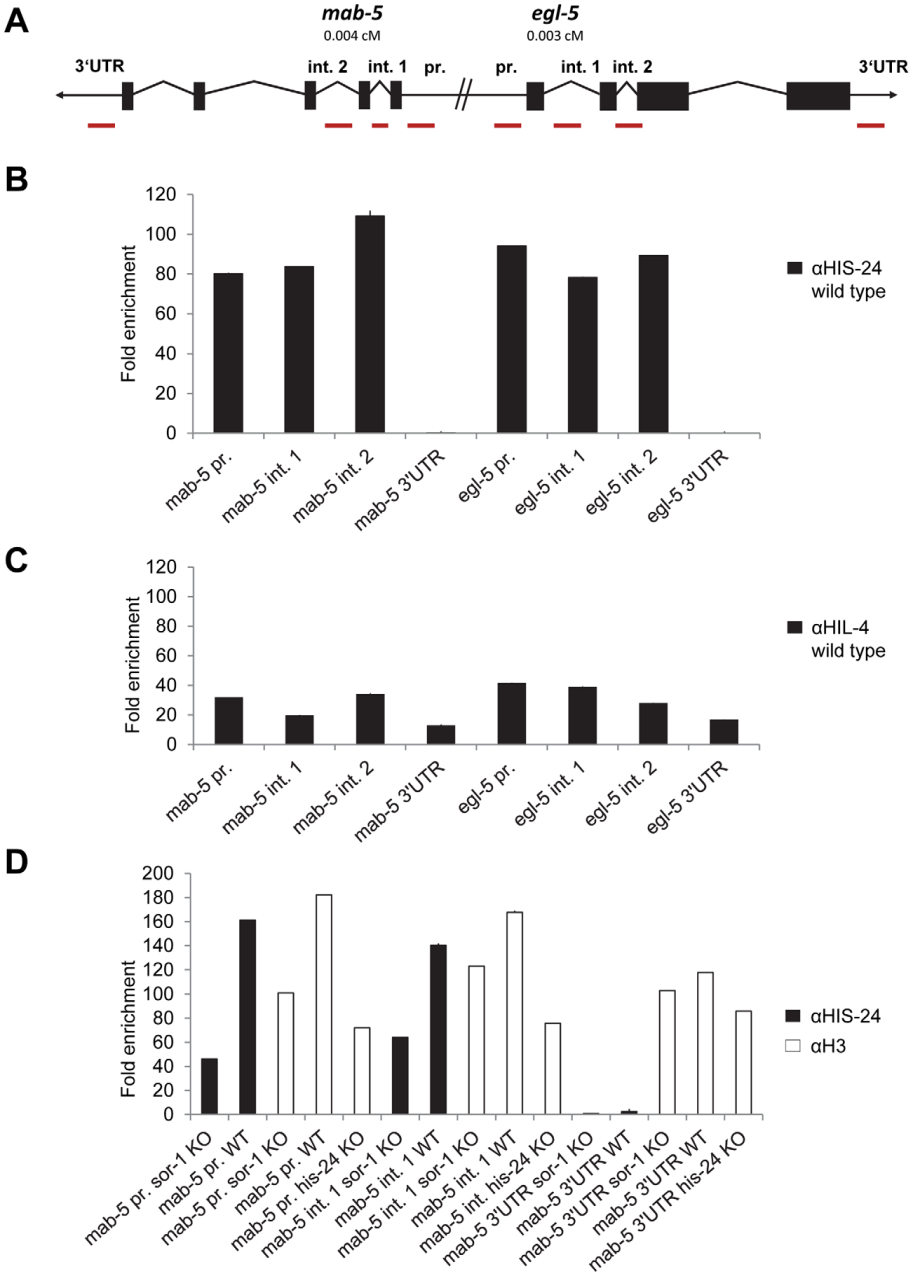
HIS-24 associates with promoters of *mab-5* and *egl-5* genes. (A) *mab-5* and *egl-5* genes are tightly clustered on chromosome III. Primer sets for qChIP-PCR are directed to the *mab-5* and *egl-5* promoters, introns and 3′UTR (red bars). (B) qChIP-PCR assay determining HIS-24 occupancy at the *mab-5*, and *egl-5* genes. The results were normalized to binding by anti-HIS-24 antibody in *his-24* mutant strains and performed in triplicates. Error bars indicate ±SD (see Table S3). (C) qChIP using anti-HIL-4 antibody and total protein isolated from wild type worms. (D) Decreased level of HIS-24 enrichment at *mab-5* using anti-HIS-24 antibody and *mab-5::gfp* transgenic strain in *sor-1* background mutation (KO) as well as wild type (WT) animals. H3 occupancy at *mab-5* loci is affected in *sor-1* and *his-24* background. In *sor-1* and *his-24* background *mab-5* is ectopically expressed in contrast to repressed *mab-5* in wild type animals. (C, D) All results were normalized to binding by control IgG antibody and performed in triplicates. Error bars indicate ±SD (see Table S3).

Since HIS-24 and HPL-2 are required for inhibiting the ectopic expression of *mab-5* and *egl-5* Hox genes, we tested if HIS-24 and HPL-2 bind directly to their promoters *in vivo* and therefore regulate *egl-5* and *mab-5* transcription. The primer sets used for quantitative ChIP-PCR (qChIP-PCR) analysis were directed to the promoters, introns and 3′UTR regions of *mab-5* and *egl-5* genes. Remarkably, *mab-5* and *egl-5* are tightly clustered on chromosome III, suggesting that chromatin structure coordinately regulates the expression of these genes (Figure 7A). qChIP-PCR analysis revealed that HIS-24 is indeed associated with the promoters and introns of *mab-5* and *egl-5* genes (Figure 7B). In contrast, we did not see any HIS-24 binding to 3′UTR regions (Figure 7B). However they are occupied by H3 (Figure 7D). As shown, the anti-HIS-24 antibody binds with higher affinity to *egl-5* and *mab-5* genes than the anti-HIL-4 antibody, which is cross-reactive to *C. elegans* linker histone variants [14] (Figure 7C). Next, to verify the specificity of the HIS-24 binding to Hox genes, we tested the HIS-24 binding to *mab-5* gene ectopically expressed in *sor-1* background mutation. As previously reported, SOR-1 (together with SOP-2) shares many structural and functional properties with the PRC1 complex, and is involved in the global repression of *egl-5* or *mab-5* Hox gene expression [25]. As shown, we detected a significantly decreased level of HIS-24 at this region compared to the situation in wild type animals, implicating that HIS-24 enables *mab-5* transcriptional repression, thereby influencing its expression (Figure 7D). Additionally, we observed lower levels of histone H3 occupancy at the *mab-5* promoter in *sor-1* background mutation than in wild type animals, suggesting that the difference in H3 levels could be due to the nucleosome free region that forms at high levels of expression (Figure 7D). In addition, *mab-5* promoter and intron regions in the *his-24* mutant animals showed decreased enrichment of the histone H3 than in wild type animals, suggesting that binding of H3 and HIS-24 can be positively correlated at regulatory regions. In comparison, the H3 changes at 3′UTR region of *mab-5* in *sor-1* and *his-24* background mutation were relatively mild than in wild type animals (Figure 7D).

Unfortunately, we have failed so far to detect HPL-2 at this region using direct ChIP approach.

### HIS-24 and HPL-2 act in parallel pathway as MES-2/-3

**Figure 8:**
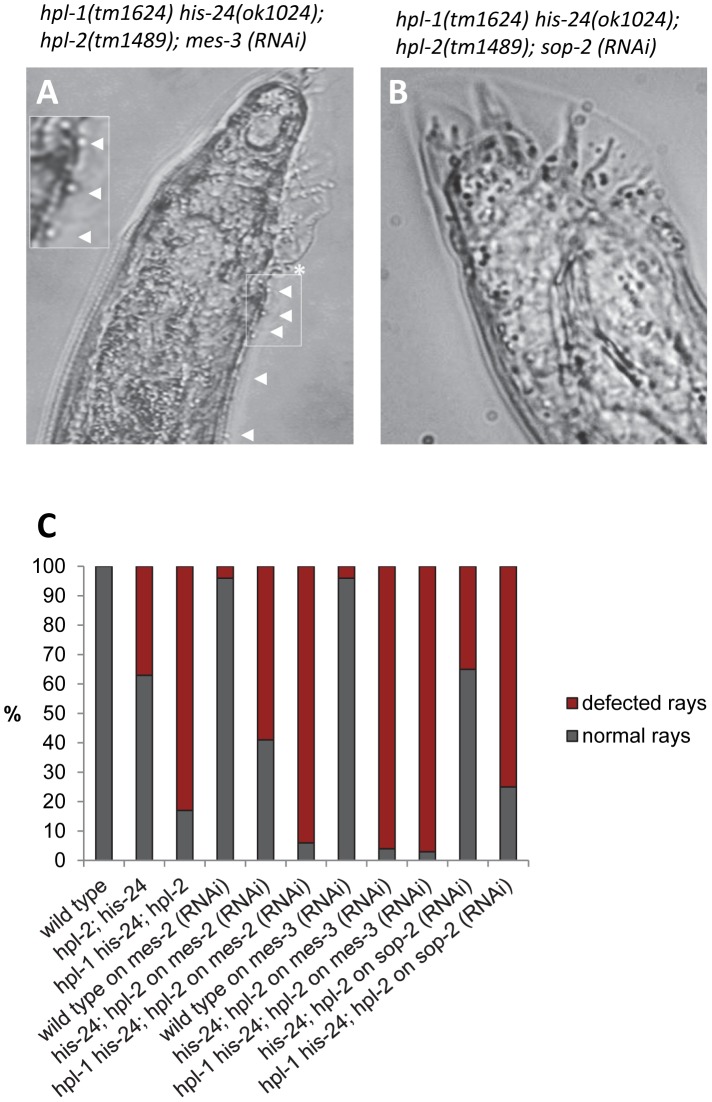
MES-2 and MES-3 enhances the number of defected rays in animals lacking *hpl-2* and *his-24*. (A) RNA interference (RNAi) of *mes-3* leads to enhancement of *hpl-2*; *hpl-1his-24* mutant male phenotype in contrast to (B) *sop-2*-depleted triple mutant animals. Ectopic rays are indicated with arrowheads and under-developed rays by a star. (C) Quantification of ray defects associated with *hpl-2; his-24* double and *hpl-2*; *hpl-1his-24* triple mutations. The animals were growing on *him-14* and *mes-2* or *mes-3* as well as *sop-2* feeding plates at 21°C. *sop-2* deletion is epistatic to *hpl-2; his-24* double mutation.

Hox genes are transcriptionally repressed by the evolutionally conserved Polycomb group (PcG) proteins through the H3K27me3 mark in a lineage specific fashion [30]–[31]. In *Drosophila*, a member of the Polycomb group (PcG), the H3K27 histone methyltransferase E(Z) has been identified as a stable repressor of Hox genes [32]. In *C. elegans*, orthologs of the PcG chromatin repressors E(Z) and ESC, namely MES-2 and MES-6 influence expression of Hox genes and male tail development [23]. Since Polycomb group (PcG) proteins (MES-2/3/6 complex) are involved in the repression of Hox genes, we performed genetic epistasis analysis of *mes-2*- and *mes-3*-depleted triple mutant animals [23], [33]. Interestingly, *hpl-2(tm1489); his-24(ok1024)* double as well as *hpl-2(tm1489); hpl-1(tm1624) his-24(ok1024)* triple mutant males on *mes-2* or *mes-3* feeding plates showed an increased number of ectopic rays (∼2-fold) and defective rays in comparison to *mes-3*- or *mes-2* - depleted double mutant males (Figure 8A, 8C; Table S2). As shown, loss of HPL and HIS-24 together with depletion of *mes-2* or *mes-3* resulted in additive defects implying that HPL and HIS-24 act in parallel pathway as MES-2 or MES-3.

We also phenotyped *hpl-2(tm1489); his-24(ok1024)* double and *hpl-2(tm1489); hpl-1(tm1624) his-24(ok1024)* triple mutant males on *sop-2* feeding plates (Figure 8B, 8C, Table S2). As previously reported, SOP-2 forms a novel PcG-like complex that may function analogously to PRC1 in *C. elegans* and regulates expression of Hox genes [25]. Homologs of SOR-1 and SOP-2 are not found in other organisms, including even the very closely related *C. briggsae* suggesting a *C. elegans* specific mechanism on an essential global gene regulatory system [25]. Remarkably, we did not observe any influence of SOP-2 depletion in the *hpl-2; his-24* double and *hpl-2; hpl-1 his-24* triple mutant background suggesting that *sop-2* appears to be epistatic to *hpl-2; his-24* deletion.

### HIS-24K14me1 and HPL-2 bind H3K27me3 chromatin mark

Recently, we have reported that HIS-24 specifically interacts with H3K27 trimethylated and H3K27 unmodified peptides [18]. While HPL-1 and HPL-2 were able to pull down native HIS-24K14me1, and HPL-2 failed to bind either modified or unmodified HIS-24 peptides *in vitro*, we asked whether HPL-2 and HIS-24K14me1 repress the transcription of *egl-5* and *mab-5* genes by binding to H3K27me3 [16], [18].

**Figure 9:**
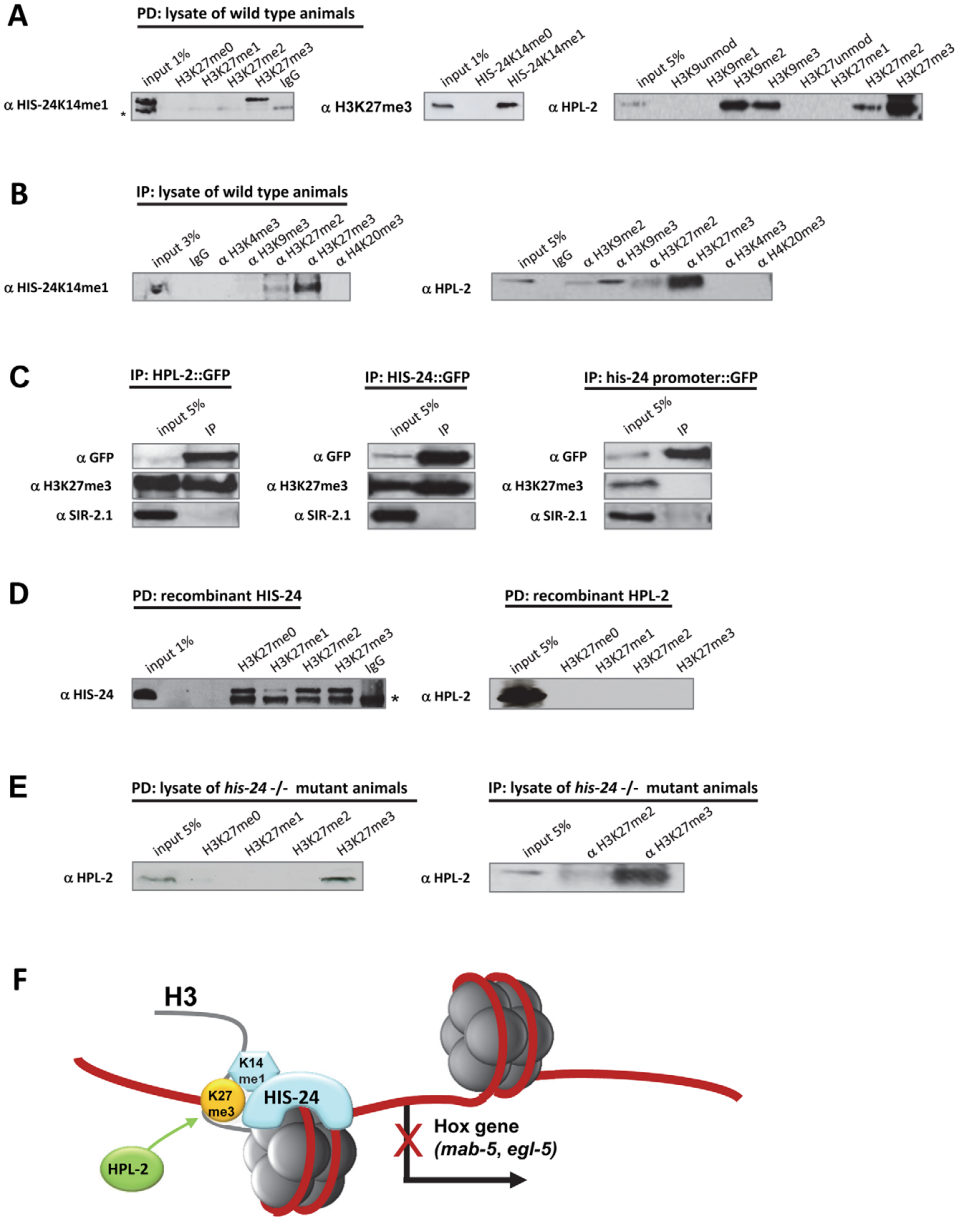
HIS-24K14me1 and HPL-2 bind to the H3K27me3 chromatin mark. (A, B) HIS-24K14me1 protein specifically recognizes H3K27me3 chromatin mark and HPL-2 binds H3K27me2/me3, and H3K9me2/me3 in peptide pulldown assay (PD) and immunoprecipitation experiment (IP). Non-specific bands are indicated by a star. (C) HPL-2::GFP and HIS-24::GFP pull down H3K27me3. In contrast, GFP under *his-24* promoter did not precipitate H3K27me3. In the bottom panel, SIR-2.1 was used as a negative control [18]. (D) Recombinant expressed HIS-24 binds H3K27me0/2/3 in contrast to HPL-2. (E) Deficiency of HIS-24 does not influence the binding of HPL-2 to H3K27me3. (F) Simplified model of HIS-24K14me1 and HPL-2 regulation of Hox gene expression. HIS-24K14me1 does not interact with HPL-2.

By peptide pull down assay (PD) we observed that HIS-24K14me1 interacts preferentially with H3K27me3 peptide when compared to the unmodified, mono- or di-methylated H3K27 peptides, and conversely, native H3K27me3 binds only the methylated form of HIS-24 peptide (Figure 9A). Furthermore, we found strong preference of HPL-2 for the trimethylated form of H3K27, as well as for H3K27me2 and H3K9me2/3 as previously reported (Figure 9A) [16]. No interaction was observed between H3K9me0/1 or H3K27me0/1. We confirmed the results obtained from peptide pull down (PD) by an immunoprecipitation (IP) approach using antibodies raised against different chromatin modification marks and lysates of wild type animals (Figure 9B). Additionally, we were able to pull down native H3K27me3 using a GFP-antibody directed against GFP-tagged HPL-2 and HIS-24 (Figure 9C). As a control we used GFP expressed protein under the his-24 promoter to demonstrate the specificity of HPL-2 and HIS-24 binding to H3K27me3 (Figure 9C). To confirm that HPL-2 and HIS-24 indeed display H3K27me3 binding, we expressed HPL-2 and HIS-24 in *E. coli*. We did not detect the interaction of HPL-2 with H3K27me3 in contrast to HIS-24, suggesting that additional factors (transcription factors, RNAi machinery, post-translational modifications of HPL-2) are involved in the mediation of HPL-2 binding to H3K27me3 (Figure 9D, 9F). In the case of HIS-24 we detected strong preference for H3K27me peptides apart from H3K27me1 (Figure 9D). The differences in the binding to H3K27me3 between bacterially expressed HIS-24 and native HIS-24 can be explained by the fact that bacterially expressed proteins are not methylated and only the methylated form of HIS-24 binds specifically the H3K27me3. Finally, to exclude that the binding of HPL-2 to H3K27me3 takes place via interaction with the *C. elegans* HIS-24, we repeated the pull downs using extracts obtained from *his-24(ok1024)* mutant animals (Figure 9E). We detected a preference of HPL-2 for H3K27me3 independently of HIS-24 however this binding was reduced compared to binding of HPL-2 to H3K27me3 in the presence of HIS-24 (Figure 9B, 9E).

### HIS-24K14me1 rescues the developmental patterning of the male tail

**Figure 10:**
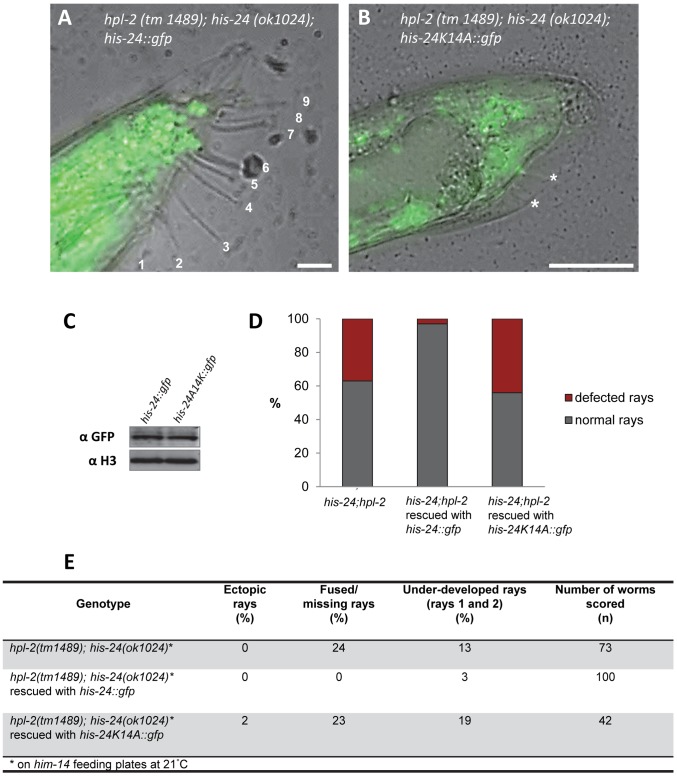
HIS-24::GFP expression in *hpl-2;his-24* double mutant animals restores normal male tail development. (A–C) The exogenous, mutated form of *his-24K14A::gfp* is expressed at the level comparable to those in animals carrying *his-24::gfp* wild type form. (A) Scale bar 10 µm. (B) Scale bar 25 µm. Stars point to under-developed rays. (D) Quantification of ray defects (E) associated with *his-24K14A::gfp* and *his-24::gfp* in *hpl-2; his-24* background mutation. The animals were growing on *him-14* feeding plates at 21°C.

To assess whether the methylated form of HIS-24 has a causal role in the observed changes of the male tail morphology, we generated *his-24::gfp* and *his-24K14A::gfp* transgenic worms in the *hpl-2(tm1489)*; *his-24(ok1024)* mutant background. We observed that the restoration of HIS-24 levels by expression of HIS-24::GFP rescued the male phenotype and the fused/missing rays were down nearly to zero in the transgenic line (Figure 10). Importantly, the nonmethylatable HIS-24K14A::GFP mutant failed to rescue the wild type rays development in *hpl-2; his-24* animals, suggesting that HIS-24 methylation at lysine 14 is necessary to regulate male tail development (Figure 10B, 10D, 10E). These results also imply that, at 21°C, *hpl-2* and *his-24* play a redundant role in the regulation of positional identity in the *C. elegans* males. Importantly, the analysis of transgene expression at the cellular level by immunostaining and immunoblotting of the rescued *hpl-2(tm1489)*; *his-24(ok1024)* animals verified that the exogenous HIS-24K14A::GFP mutated form was expressed at a level comparable to that in animals carrying HIS-24::GFP wild type form (Figure 10C).

## Discussion

HP1 and H1 are heterochromatin components that are believed to be associated with global repression of transcriptional activity 4–5. Surprisingly, our microarray analysis showed that H1 and HP1 play more dynamic and gene-specific roles in the roundworm *C. elegans*. They grossly affect only a few genes and can have an overlapping function in the same or parallel pathways where they regulate common target genes.

In particular, we found that HIS-24 and HPL-2 can regulate a shared target, the Hox genes. Although, the *C. elegans* homeobox genes (*egl-5*, *mab-5*) are silenced by mechanisms involving H3K27 trimethylation, we showed that the methylated form of HIS-24 and HPL-2 can also serve as essential protein components in establishing and/or maintaining the repressive chromatin state at the selected Hox genes, presumably through their binding to H3K27me3 (Figure 9F).

### Effect of HIS-24 and HPL on gene expression profile and chromatin organization

Our microarray analyses support a role of H1 and HP1 in specific gene regulation, rather than a general repressive function [34]–[36]. Despite global changes in chromatin compaction and synergism of HIS-24 and HPL in aspects of many developmental processes we observed very few and slight changes in gene expression profile of mutants when compared with wild type animals. We detected a set of shared up- and down-regulated genes by HIS-24 and HPL suggesting that redundant roles for HIS-24 and HPL also exist. The relatively small number of regulated genes in observed triple mutant animals may indicate that HPL proteins and HIS-24 serve to fine-tune the regulation of key genes during development or differentiation. This model can be explained by the fact that the sequential arrangement of the linker histone HIS-24 and HPL-2 on the chromatin fibre might influence higher-order chromatin structure and effect nucleosome positioning, and stability [36]. It is possible that the different HPL subtypes and HIS-24 confer subtle differences in the properties of the chromatin fiber which allow for quantitative modulation of gene expression [34], [35]. Although the changes in gene transcription are subtle, we think that even 1.5-fold differences in expression can contribute to the marked phenotypic consequences we observed.

### Model of transcriptional regulation of *egl-5* and *mab-5* by HPL-2 and HIS-24K14me1

We demonstrated that HIS-24K14me1, together with HPL-2, has a causal role in transcriptional silencing of *egl-5* and *mab-5*. We propose that HPL-2 and HIS-24K14me1 may serve as essential protein components in establishing and/or maintaining the repressive chromatin state at the selected Hox genes through their interactions with H3K27me3. While we did not observe any phenotypic effects on male tail development either in *hpl-2; hpl-1* nor in *hpl-1 his-24* background, we speculate that HPL-2 acts redundantly with HIS-24K14me1 to regulate the positional identity in the *C. elegans* males. Loss of the two heterochromatin components, HIS-24K14me1 and HPL-2, causes significant changes in chromatin structure affecting Hox gene expression in *C. elegans*. However, since no interaction of HPL-2 and HIS-24K14me1 was observed in immunoprecipitation experiments, it is possible that HPL-2 together with HIS-24K14me1 might be a part of the same protein group involved in the regulation of Hox gene expression. The high degree of redundancy between *his-24* and *hpl-2* in Hox gene regulation might indicate that these two proteins are the only readers acting in parallel to perform the same role in translating the effects of histone H3K27 trimethylation. However, since we have failed so far to detect HPL-2 at the Hox gene region using direct ChIP approach, it is possible that the mechanisms by which HPL-2 regulates *mab-5* and *egl-5* might be indirect, involve intermediate factors (RNAi machinery, transcription factors) and depend on an architectural level in the cellular context.

### HIS-24K14me1 and HPL-2 as a part of the PcG silencing complex

In mammals, H1 regulates Hox gene activation by promoting DNA demethylation [13]. Although *C. elegans* does not possess methylated DNA, we speculate that H1 can still influence Hox gene regulation and, together with HPL-2, regulate Hox gene expression as a part of the PcG silencing complex. The interaction of HPL-2 and HIS-24K14me1 with H3K27me3 can regulate the Hox gene in parallel pathway as MES-2 or MES-3, and can be directed to specific parts of the genome. Notably *C. elegans* HP1/HPL-2 does not follow the H3K9me2/me3 code [37]–[41] but it is sufficient to recognize, and to bind H3K27me2/me3. Remarkably, HIS-24 is required for optimal HPL-2 binding to H3K27me3 *in vivo*.

Interestingly, some PcG proteins containing a chromodomain similar to that found in *C. elegans* HPL-2 and mammalian HP1s have been shown to bind H3K27me3 [30], [42].

Overall, these and our previous results implicate that HPL and HIS-24 share some common functions even though there are differences among these proteins [16]–[17], [26]. We conclude that a combination of the H3K27me3 methylation mark, HPL-2 and HIS-24K14me1 could be a major factor in the establishment of stable patterns of selected homeotic gene expression.

## Methods

### Strains

Nematodes strains were cultured and genetically manipulated as previously described [43]. The Bristol strain (N2) was used as wild type. The following strains, obtained from the Caenorhabditis Genetics Center (CGC), were used in this study: *his-24(ok1024)*X, *hil-3(ok1556)*X (both strains outcrossed 1×), *hpl-1(tm1624)*X (outcrossed 4×), *hpl-2(tm1489)*III (outcrossed 4×). Transgenic strain (transcriptional reporter) expressing GFP under the control of the his-24 promoter was kindly provided by BC *C. elegans* Gene Expression Consortium, Canada. The double mutants *hpl-1(tm1624)*X *his-24(ok1024)*X, *hpl-2(tm1489)*III; *his-24(ok1024)*X, *hpl-2(tm1489)*III; *hil-3(ok1556)*X and the triple mutant strain *hpl-2(tm1489)*III; *hpl-1(tm1624)*X *his-24(ok1024)*X were generated by crossing. *his-24::gfp* (stable integrated EC602 strain [26]) and *his-24K14A::gfp* transgenic strains were crossed with the *hpl-2(tm1489)*III; *his-24(ok1024)*X. The generation of *his-24K14A::gfp* transgenic strain was previously described [16].

For the reporter gene analysis following transgenic strains: EM599 [*egl-5::gfp*; *him-5(e1490)*V; *lin-15B(n765)*X; *bxIs13*], OP27 [*unc-119(ed3)*III; *wgIs27*], OP54 [*unc-119(ed3)*III; *wgIs54*] and HZ111 [*mab-5::gfp*; *muIs16* II; *sor-1(bp1)/qC1 dpy-19(e1259) glp-1(q339)*III; *him-5(e1490)*V], kindly provided by CCG, were used.

The brood size was scored as previously described [14].

All *C. elegans* strains were maintained at 15°C or at 21°C, unless otherwise specified.

### Protein extraction, purification, and identification

*C. elegans* H1 extraction was performed as previously described [16].

### Immunofluorescence analysis

Worms from wild type strain and the mutant worms were fixed and stained as previously described [26]. Gonads of worms were stained with fluorescent dye 4′,6′-diamidino-2-phenylindole (DAPI) diluted 1∶1000. The slides were mounted with Vectashield Mounting Medium and analyzed by using Leica DMI 6000 microscope.

### Microarray analysis

Microarray analysis from two biological replicates was performed as previously described [16], [44]. In brief, 80 to 100 animals in L4 larval stage raised at 21°C were used. The gene expression fold change was calculated from the duplicate microarray data. The fold change cut-off was 1.5 from 2 biological replicates.

### Analysis of ray phenotypes

Abnormalities of rays were identified in single, double and triple mutant males in comparison to the wild type worms. Animals were transferred on agar pads (2% agarose) and examined with differential interference contrast (DIC), using Leica DMI 6000 microscope. The number of rays, their position in relation to the anterior-posterior body axis and their shape served as basics of the analysis. Rays which were found outside of their normal formation region were defined as ectopic.

### RNA interference (RNAi) experiments

RNAi feeding experiment was performed in 50 mm NGM feeding plates (NGM plates with 100 µg/ml ampicillin, 1 mM IPTG). *him-14* (RNAi) (CGC, USA), *hpl-1* (RNAi), *mes-2* (RNAi) and *mes-3* (RNAi) bacterial clones (Sanger Institute, UK) were grown overnight at 37°C in LB medium with 100 mg/ml ampicillin and were spotted onto 50 mm NGM plates. Mixed stage L3 and L4 mutant larval worms were transferred onto feeding plates and incubated at 21°C through several generations. Males were examined on the agar pads using Leica DMI 6000 microscope. Male progeny were scored for the presence of ectopic, under-developed rays and/or ray fusions.

### Analysis of EGL-5::GFP and MAB-5::GFP expression in the single, double, and triple mutant strains

L3 stage and adult animals from each line were mounted on the agar pads and examined under Leica DMI 6000 microscope. Males were scored for the presence of ectopic EGL-5::GFP or MAB-5::GFP expression.

### Chromatin immunoprecipitation

ChIP was performed as previously described [45] with several modifications. Mixed stage L4 and adult worms were homogenized in ice-cold FA lysis buffer (50 mM HEPES/KOH pH 7.5, 1 mM EDTA, 1% Triton X-100, 0.1% sodium deoxycholate; 150 mM NaCl) with complete protease inhibitor cocktail (Roche) and 1% Triton X-100 using liquid nitrogen. Worm lysate was sonicated with a Branson Digital Sonifier using following settings: 30% amplitude for 3 min total. Protein concentration of the extract was determined by the Coomassie Plus (Bradford) Protein Assay. Worm extract was incubated with the following antibodies: anti-H3 (Abcam 1791), anti-H3K27me2 (Upstate 07-322), anti-H3K27me3 (kindly provided by T. Jenuwein), anti-H3K9me2 (Abcam 1220), anti-H3K4me3 (Abcam 1012), anti-GFP (Roche) and anti-HIS-24. Proteins were immunoprecipitated using G-agarose beads (Pierce). *mab-5* and *egl-5* genes were detected by qPCR using iCycler iQ™ Multi- Color real time PCR detection system (Bio-Rad). Primer sequences are available on request.

### Peptide pull down analysis

Peptide pull downs were performed as previously described [46]. 10 µg of each biotinylated peptide was coupled to streptavidin- agarose beads (Pierce). For peptide binding experiments following peptides were used: H3 mono-, di- or trimethylated at K9, H3 mono-, di- or trimethylated at K27, H3 unmethylated at K27, HIS-24 monomethylated at K14 and HIS-24 unmethylated at K14. Peptides were generated by Squarix (Germany). Worm extracts were incubated for 2 h at 4°C with the beads (constant rotation). Beads were washed six times with PD 150 buffer (20 mM Hepes pH 7.9, 150 mM KCl, 0.2% Triton-X 100, complete protease inhibitor cocktail, 20% glycerol). Bounded proteins were separated on gradient NuPAGE SDS gel (4–12%).

### Western and dot blot

*C. elegans* lysates were prepared and analyzed by western blot as previously described [16], [18].

### Immunoprecipitation

Mixed populations of L4 worms carrying the *hpl-2::gfp* transgene or wild type worms were homogenized [47]. About 1.5 mg of total precleared protein was incubated with following antibodies GFP-Trap^R^ –A beads (Chromotek, Germany), anti- H3 (Abcam 1791), anti-H3K27me2 (Upstate 07-322), anti-H3K27me3 (kindly provided by T. Jenuwein), anti-H3K9me2 (Abcam 1220), anti-H3K4me3 (Abcam 1012), anti-H3K9me3 (Abcam 8898) or anti-H4K20me3 (Abcam 9053) at 4°C overnight. Next, the complexes were washed six times with PD150 buffer for 5 minutes at 4°C (20 mM Hepes, pH 7.9; 150 mM KCl, 0.2% Triton X-100, 1× Protease Inhibitor (Roche), 20% glycerol). Finally, the immunoprecipitated proteins were resolved on NuPAGE SDS gradient gel (4–12%) and western blotted with antibodies against H3K27me3 (1∶20 000 dilution), HPL-2 (1∶2000 dilution; kindly provided by F. Palladino), HIS-24K14me1 (1∶10000 dilution) and GFP (Roche; 1∶20000 dilution).

### Expression of recombinant HPL-2 protein

The pGEX HPL-2a plasmid (kindly provided by F. Palladino) and HIS-24 pet3a plasmid were expressed in *E. coli* BL21(DE3) and the recombinant proteins were used for the peptide pull down assay.

### Accession numbers

The microarray data can be found in the Gene Expression Omnibus (GEO) of NCBI through accession number GSE33339.

## Supporting Information

Figure S1 The levels of heterochromatin marks are not altered in the *hpls*, *his-24* mutant animals. No changes of the H3K27me3, H3K9me3 and H3 levels were observed in single, double and triple mutant animals.
(PDF)

Figure S2 Reduced level of HPL-1 after depletion. Reduction of HPL-1 level in *hpl-1* depleted *his-24; hpl-2* double mutant animals in contrast to *his-24; hpl-2* double, where HPL-1 is present.
(PDF)

Table S1 Differentially co-expressed genes for L4 stage larvae in *hpl-2; hpl-1* and *his-24 hpl-1; hpl-2* compared to wild type (FDR<0.05). Each table represents the following ontologies: Biological Process (BP) and/or Cellular Component (CC). Gene Ontology (GO) terms taken from WormBase (http://www.wormbase.org). n/a –not available.
(XLS)

Table S2 Ray defects associated with *hpl-1*, *hpl-2* or *his-24* mutations.
(DOC)

Table S3 Standard deviation of qChIP-PCR analysis for Figure 7.
(DOC)
