# Supplementary figures and images for: Transcriptional Repression of Hox Genes by C. elegans HP1/HPL and H1/HIS-24
Source: PLoS Genet. 2012 Sep 13;8(9):e1002940. doi: 10.1371/journal.pgen.1002940 (PMC3441639; doi:10.1371/journal.pgen.1002940)

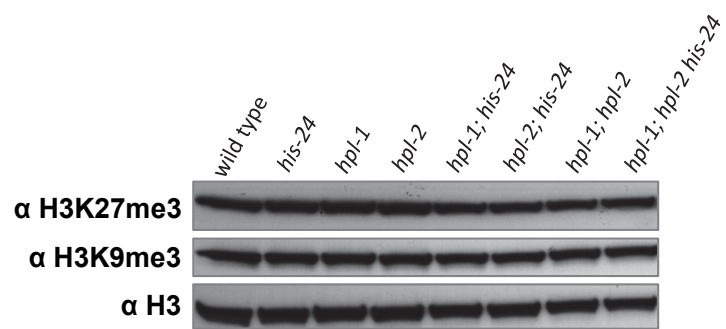

Supplement: Figure S1 — The levels of heterochromatin marks are not altered in the hpls, his-24 mutant animals. No changes of the H3K27me3, H3K9me3 and H3 levels were observed in single, double and triple mutant animals. (PDF) [file pgen.1002940.s001.pdf]

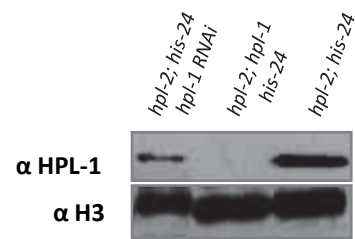

Supplement: Figure S2 — Reduced level of HPL-1 after depletion. Reduction of HPL-1 level in hpl-1 depleted his-24; hpl-2 double mutant animals in contrast to his-24; hpl-2 double, where HPL-1 is present. (PDF) [file pgen.1002940.s002.pdf]
