# Supplementary material for: Transcriptional Repression of Hox Genes by C. elegans HP1/HPL and H1/HIS-24
Source: PLoS Genet. 2012 Sep 13;8(9):e1002940. doi: 10.1371/journal.pgen.1002940 (PMC3441639; doi:10.1371/journal.pgen.1002940)
Supplement: Table S2 — Ray defects associated with hpl-1, hpl-2 or his-24 mutations. (DOC) [file pgen.1002940.s004.doc]

**Table S2. Ray defects associated with *hpl-1*, *hpl-2* or *his-24*** mutations.

| **Genotype** | **Ectopic rays (%)** | **Fused/missing rays (%)** | **Under-developed rays (rays 1 and 2) (%)** | **Number of worms scored (n)** |
| --- | --- | --- | --- | --- |
| wild type* | 0 | 0 | 0 | 72 |
| *his-24(ok1024)** | 0 | 0 | 0 | 54 |
| *hil-3(ok1556)** | 0 | 0 | 0 | 31 |
| *hpl-1(tm1624)** | 0 | 0 | 0 | 48 |
| *hpl-2(tm1489)** | 0 | 0 | 0 | 67 |
| *hpl-2(tm1489); his-24(ok1024)** | 0 | 24 | 13 | 73 |
| *hpl-2(tm1489); hil-3(ok1556)** | 0 | 0 | 0 | 78 |
| *hpl-1(tm1624) his-24(ok1024)** | 0 | 0 | 0 | 54 |
| *hpl-1(tm1624) his-24(ok1024); hpl-2(tm1489)** | 4 | 37 | 42 | 107 |
| *hpl-1(tm1624); hpl-2(tm1489)** | 0 | 0 | 0 | 58 |
| wild type on *mes-2* feeding*** | 3 | 1 | 0 | 86 |
| *his-24(ok1024); hpl-2(tm1489)* on *mes-2* feeding*** | 5 | 23 | 31 | 82 |
| *hpl-1(tm1624) his-24(ok1024); hpl-2(tm1489)* on *mes-2* feeding*** | 10 | 43 | 41 | 102 |
| wild type on *mes-3* feeding*** | 5 | 1 | 0 | 85 |
| *his-24(ok1024); hpl-2(tm1489)* on *mes-3* feeding*** | 12 | 44 | 40 | 96 |
| *hpl-1(tm1624) his-24(ok1024); hpl-2(tm1489)* on *mes-3* feeding*** | 9 | 45 | 46 | 107 |
| *hpl-2(tm1489); his-24(ok1024)* on *sop-2* feeding*** | 0 | 21 | 14 | 95 |
| *hpl-1(tm1624) his-24(ok1024); hpl-2(tm1489)* on *sop-2* feeding*** | 3 | 35 | 37 | 101 |
| *** on *him-14* feeding plates at 21°C** | | | | |
