# Supplementary material for: Transcriptional Repression of Hox Genes by C. elegans HP1/HPL and H1/HIS-24
Source: PLoS Genet. 2012 Sep 13;8(9):e1002940. doi: 10.1371/journal.pgen.1002940 (PMC3441639; doi:10.1371/journal.pgen.1002940)
Supplement: Table S3 — Standard deviation of qChIP-PCR analysis for Figure 7. (DOC) [file pgen.1002940.s005.doc]

**Table S3. Standard deviation of qChIP-PCR analysis**

**HIS-24 binding to the regulatory regions of the *mab-5* and *egl-5 Hox* gene.**

| **Gene region** | **Standard deviation**  **±SD** |
| --- | --- |
| *mab-5* promoter | 0,60 |
| *mab-5* intron 1 | 0,12 |
| *mab-5* intron 2 | 2,64 |
| *mab-5* 3’UTR | 0,82 |
| *egl-5* promoter | 0,08 |
| *egl-5* intron 1 | 0,40 |
| *egl-5* intron 2 | 0,24 |
| *egl-5* 3’UTR | 0,84 |

**HIL-4 binding to the regulatory regions of the *mab-5* and *egl-5 Hox* gene**.

| **Gene region** | **Standard deviation**  **±SD** |
| --- | --- |
| *mab-5* promoter | 0,04 |
| *mab-5* intron 1 | 0,24 |
| *mab-5* intron 2 | 0,40 |
| *mab-5* 3’UTR | 0,72 |
| *egl-5* promoter | 0,24 |
| *egl-5* intron 1 | 0,24 |
| *egl-5* intron 2 | 0,08 |
| *egl-5* 3’UTR | 0,08 |

**HIS-24 and H3 binding to the regulatory regions of the *mab-5 Hox* gene in *mab-5::gfp* strain in *sor-1* background. H3 binding to the regulatory regions of the *mab-5* foci in *his-24* background.**

| **Gene region** | **Standard deviation**  **±SD** |
| --- | --- |
| *mab-5* pr. *sor-1* KO (αHIS-24) | 0,08 |
| *mab-5* pr. WT (αHIS-24) | 0,18 |
| *mab-5* pr. *sor-1* KO (αH3) | 0,24 |
| *mab-5* pr. WT (αH3) | 6,31E-30 |
| *mab-5* pr. *his-24* KO (αH3) | 0,02 |
| *mab-5* int. 1 *sor-1* KO (αHIS-24) | 0,02 |
| *mab-5* int. 1 WT (αHIS-24) | 1,44 |
| *mab-5* int. 1 *sor-1* KO (αH3) | 0,01 |
| *mab-5* int. 1 WT (αH3) | 1,44 |
| *mab-5* int. *his-24* KO (αH3) | 0,02 |
| *mab-5* 3'UTR *sor-1* KO (αHIS-24) | 0,12 |
| *mab-5* 3'UTR WT (αHIS-24) | 2,20 |
| *mab-5* 3'UTR *sor-1* KO (αH3) | 0,13 |
| *mab-5* 3'UTR WT (αH3) | 0,24 |
| *mab-5* 3'UTR *his-24* KO (αH3) | 0,01 |
